# Comparative Predictive Value of First-Trimester Crown–Rump Length and Nuchal Translucency Discordance for Fetal Growth Restriction in Twin Pregnancies: A Retrospective Cohort Study

**DOI:** 10.3390/jcm15083129

**Published:** 2026-04-20

**Authors:** Cansın Eroğlu, Ömer Osman Eroğlu, Ali Turhan Çağlar

**Affiliations:** Department of Obstetrics and Gynecology, Ankara Etlik City Hospital, 06010 Ankara, Turkey; omerosmaneroglu@gmail.com (Ö.O.E.); turhan_caglar@yahoo.com (A.T.Ç.)

**Keywords:** twin pregnancy, crown–rump length, nuchal translucency, fetal growth restriction, first-trimester screening, discordance

## Abstract

**Background/Objectives:** Twin pregnancies carry substantially elevated perinatal risks, yet tools for first-trimester risk stratification remain limited. This retrospective cohort study evaluated the predictive value of crown–rump length (CRL) and nuchal translucency (NT) discordance for adverse perinatal outcomes in 184 twin pregnancies at Ankara Etlik City Hospital, Turkey (October 2022–January 2024). **Methods:** CRL discordance ≥ 10% and NT discordance ≥ 20% were assessed for a birth-weight-based proxy of fetal growth restriction (FGR), preeclampsia, and neonatal outcomes using multivariable logistic regression adjusted for chorionicity, body mass index (BMI), and conception mode. **Results:** CRL discordance ≥ 10% was independently associated with the birth-weight-based FGR proxy (adjusted odds ratio [OR] 7.79, 95% confidence interval [CI] 3.95–20.12, *p* < 0.001; area under the curve [AUC] 0.736). NT discordance ≥ 20% was also independently associated with the birth-weight-based FGR proxy (OR 3.74, 95% CI 1.91–8.39, *p* < 0.001; AUC 0.612). Both parameters were associated with lower Apgar scores. IVF conception was independently associated with preeclampsia in an exploratory analysis (OR 5.31, 95% CI 1.41–28.66, *p* = 0.016). Continuous modelling confirmed a dose–response relationship for CRL discordance (OR per 1% increase = 1.20, 95% CI 1.13–1.32). **Conclusions:** These findings suggest that first-trimester CRL discordance may provide useful early prognostic information for birth-weight-based adverse growth outcome in twin pregnancies, pending prospective validation in cohorts with Doppler-based FGR ascertainment.

## 1. Introduction

Although twin pregnancies account for approximately 2–3% of all births, they disproportionately contribute to perinatal morbidity and mortality due to complications specific to multiple pregnancies, such as FGR, preterm birth, and twin-to-twin transfusion syndrome (TTTS) [1,2]. Advanced maternal age and the widespread use of assisted reproductive technologies (ART) have significantly increased the incidence of twin pregnancies in recent decades; this epidemiological shift has further highlighted the clinical importance of identifying high-risk twin pregnancies at the earliest possible gestational age [3,4].

First-trimester ultrasound provides essential information for twin pregnancy management; chorionicity determination and early growth assessment are central to risk stratification and surveillance planning. CRL measurement performed between 11 and 14 weeks of gestation is the most accurate method for dating singleton pregnancies and is recommended as an early indicator of fetal growth in twin pregnancies [5,6]. CRL discordance between twins reflects early differences in placental perfusion or genetic growth potential; it has been reported to predict birth weight discordance in subsequent trimesters, selective fetal growth restriction (sFGR), and adverse perinatal outcomes, particularly in monochorionic–diamniotic (MCDA) twins [7,8]. Similarly, the NT value routinely measured for aneuploidy screening may also provide additional prognostic information beyond chromosomal abnormalities; NT discordance has been proposed as an early sign of unequal placental sharing in monochorionic pregnancies [9,10].

Despite all these observations, the independent predictive value of CRL and NT discordance on FGR has not been systematically defined in the current cohort after adjusting for established confounders such as chorionicity, mode of conception, and maternal BMI. Although studies addressing the relationship between CRL discordance and twin pregnancy outcomes exist in the current literature, prospective and comparative cohort studies evaluating both CRL and NT discordance simultaneously while adjusting for chorionicity and conception mode remain scarce [11], and no universally accepted threshold for routine clinical implementation has been established. In previous twin pregnancy studies, a CRL discordance threshold of 10% has been the most commonly used cutoff for identifying clinically relevant early intertwin growth discrepancy, particularly in relation to selective fetal growth restriction and adverse perinatal outcomes [7,12,13]. Likewise, an NT discordance threshold of 20% has been widely adopted in the twin pregnancy literature as a pragmatic marker of early intertwin discrepancy in placental sharing and hemodynamic adaptation [6,9,10]. In this study, the predictive performance of first-trimester CRL discordance ≥ 10% and NT discordance ≥ 20% for a birth-weight-based proxy of fetal growth restriction and adverse neonatal outcomes was assessed in a retrospective cohort of twin pregnancies, with preeclampsia prediction as a secondary outcome.

## 2. Materials and Methods

### 2.1. Study Design and Population

This retrospective cohort study was conducted at the Ankara Etlik City Hospital Obstetrics and Gynecology Clinic, a high-volume tertiary referral center in Turkey. All consecutive twin pregnancies followed at our clinic between October 2022 and January 2024 were eligible for inclusion in the study. The study was approved by the Ethics Committee (AEŞH-BADEK-2024-857, 25 September 2024) and conducted in accordance with the principles of the Declaration of Helsinki. Given the retrospective nature of the study, the requirement for informed consent was waived.

Inclusion criteria are as follows: twin pregnancy, first-trimester ultrasound performed between 11 and 14 weeks of gestation in a single session including both CRL and NT measurements, and availability of complete obstetric and neonatal outcome data. Exclusion criteria were defined as follows: major fetal structural anomaly (*n* = 3), chromosomal abnormality (*n* = 2), and missing records (*n* = 7). Of the 196 consecutive twin pregnancies assessed during the study period, the final analytical cohort comprised 184 twin pregnancies. Given the very small number of monochorionic–monoamniotic (MCMA) pregnancies in the dataset (*n* = 2), these cases were retained in the cohort as both had complete first-trimester and neonatal outcome data and no major structural anomaly; their inclusion was considered unlikely to materially influence the overall findings, and chorionicity was adjusted for in all multivariable models.

### 2.2. Ultrasonography and Biometric Discordance

All first-trimester ultrasound examinations were performed by certified sonographers using a GE Voluson E8 ultrasound device (GE Healthcare, Milwaukee, WI, USA) in accordance with the Fetal Medicine Foundation (FMF) protocol [14]. CRL was measured by placing a caliper between the vertex and rump while the fetus was in the neutral position. NT was measured by placing the caliper on the inner borders of the nuchal area while the fetus was in the neutral position and in the mid-sagittal section. The larger value was taken as the reference for both parameters. CRL discordance was calculated using the formula [(larger CRL − smaller CRL)/larger CRL] × 100, and a threshold of ≥10% was defined as significant [5,7]. NT discordance was calculated using the same formula, but the threshold was defined as ≥20% [9]. Although all measurements were performed by a limited number of certified operators following a standardized FMF protocol, formal interobserver and intraobserver reliability testing was not performed as part of this retrospective study. Chorionicity was determined based on the presence of the T sign (monochorionic) or the lambda (double peak) sign (dichorionic) at 11–14 weeks [10,15].

### 2.3. Outcome Definitions

The primary outcome was fetal growth restriction (FGR), operationally defined as birth weight below the 10th percentile for gestational age and sex according to INTERGROWTH-21st standards in at least one twin [16]. This birth-weight-based proxy was selected because Doppler-based antenatal growth restriction criteria were not systematically available in this retrospective dataset; this definition was applied consistently across the full cohort to ensure comparability. Secondary outcomes were: preeclampsia (new-onset hypertension ≥ 140/90 mmHg after 20 weeks of gestation with proteinuria or end-organ involvement, per ACOG criteria [17]); gestational hypertension; TTTS (Quintero stage ≥ I); intrauterine fetal death (IUFD); gestational age at delivery; mode of delivery; birth weight; and 1- and 5-min Apgar scores (recorded as continuous variables).

### 2.4. Statistical Analysis

Continuous variables were examined for normality using the Kolmogorov–Smirnov test; where appropriate, they were presented as mean ± standard deviation or median (interquartile range). For intergroup comparisons, the independent samples *t*-test or Mann–Whitney U test was used for continuous variables, and the chi-square or Fisher’s exact test was used for categorical variables. Binary logistic regression was applied to determine independent predictors of FGR and preeclampsia. Candidate variables with *p* < 0.10 in univariate analysis were included in multivariate models; multicollinearity between CRL and NT discordance required separate models for these two parameters. Odds ratios (OR) were presented with 95% confidence intervals (CI). Discriminative ability was assessed using the Receiver Operating Characteristic (ROC) curve; optimal cutoff values were determined using the Youden index. All analyses were performed using IBM SPSS Statistics version 27.0 (IBM Corp., Armonk, NY, USA); *p* < 0.05 was considered statistically significant. The number of events per variable (EPV) for the primary FGR model was 13.0 (52 events across 4 predictor variables), satisfying the conventional minimum threshold of 10. The preeclampsia model yielded an EPV of 1.8 (9 events across 5 variables), below the recommended threshold; this analysis was therefore treated as exploratory throughout all sections of this manuscript. This study was reported in accordance with the strobe guidelines (Table S1).

## 3. Results

### 3.1. Study Population

Of the 196 consecutive twin pregnancies evaluated during the study period, 184 met all inclusion criteria and formed the final cohort. The 12 excluded cases comprised three cases with major fetal structural anomaly, two with chromosomal abnormality, and seven with incomplete first-trimester or neonatal outcome records; excluded cases represented 7.1% of the initial cohort. Formal comparison of excluded versus included cases was not possible due to incomplete data, and residual selection bias cannot be excluded. The mean maternal age was 29.4 ± 6.2 years, and the mean BMI was 30.8 ± 5.4 kg/m^2^. The cohort comprised 133 dichorionic–diamniotic (DCDA, 72.3%), 49 monochorionic–diamniotic (MCDA, 26.6%), and 2 monochorionic–monoamniotic (MCMA, 1.1%) pregnancies; MCMA cases were retained given complete data availability and absence of structural anomalies, and chorionicity was adjusted for in all multivariable models. IVF conception was recorded in 23.9% of cases (*n* = 44), and nulliparity in 56.5% (*n* = 104). Demographic and clinical characteristics are presented in Table 1.

Overall, CRL discordance ≥ 10% was detected in 33.7% of cases (*n* = 62), while NT discordance ≥ 20% was detected in 39.7% of cases (*n* = 73). The prevalence of FGR in at least one twin was 28.3% (*n* = 52), preeclampsia 4.9% (*n* = 9), gestational hypertension 9.2% (*n* = 17), TTTS 6.0% (*n* = 11), and IUFD 2.2% (*n* = 4).

### 3.2. CRL Discordance and Perinatal Outcomes

In pregnancies with CRL discordance ≥ 10%, the FGR rate was 53.2% (*n* = 33), while in those with CRL discordance < 10% it was 15.6% (*n* = 19) (univariate OR 6.57, 95% CI 3.28–13.16, *p* < 0.001). In the multivariate analysis adjusted for chorionicity, BMI, parity, and IVF status, CRL discordance ≥ 10% remained a strong and independent predictor of FGR (OR 7.79, 95% CI 3.95–20.12, *p* < 0.001). Chorionicity was also independently associated with the birth-weight-based FGR proxy (monochorionic vs. dichorionic OR 4.38, 95% CI 1.88–10.22, *p* < 0.001). These findings are summarized in Table 2.

To further characterize this association, CRL discordance was also modelled as a continuous variable in the multivariable logistic regression (adjusted for chorionicity, IVF status, BMI, and parity): each 1% increment in CRL discordance was independently associated with a 20% increase in the odds of FGR (OR per 1% increase = 1.20, 95% CI 1.13–1.32), confirming a dose–response relationship rather than a mere threshold effect. In contrast, the NT discordance modelled as a continuous variable did not reach statistical significance (OR per 1% increase = 1.02, 95% CI 0.99–1.05), further supporting the superior predictive value of CRL discordance.

**Figure 1:**
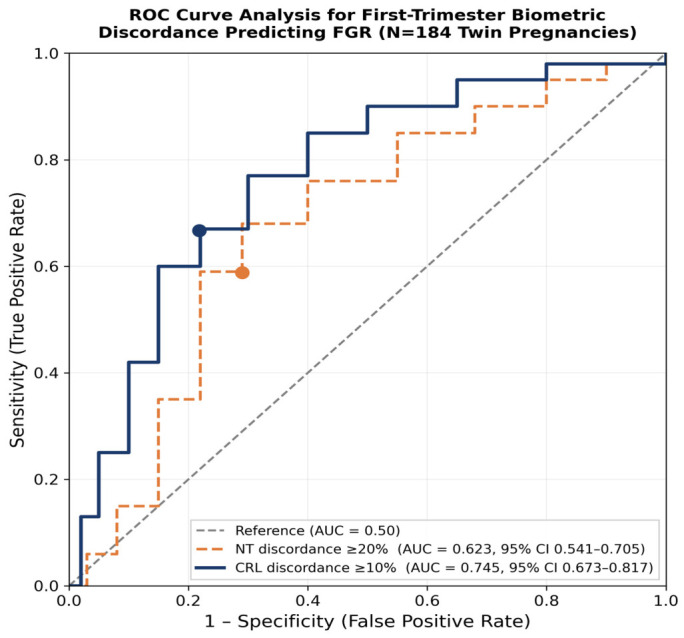
Receiver operating characteristic (ROC) curves for CRL discordance and NT discordance in predicting fetal growth restriction in at least one twin. CRL discordance showed better discrimination than NT discordance (AUC 0.736, 95% CI 0.673–0.817 vs. AUC 0.612, 95% CI 0.541–0.705). The optimal cutoff for CRL discordance was 9.9% (sensitivity 65.4%, specificity 78.0%); the optimal cutoff for NT discordance was 21.4% (sensitivity 57.7%, specificity 70.8%). The diagonal reference line represents non-discrimination (AUC 0.50). Abbreviations: AUC, area under the curve; CI, confidence interval; CRL, crown–rump length; FGR, fetal growth restriction; NT, nuchal translucency.

ROC analysis showed that CRL discordance demonstrated good discriminatory ability for FGR (AUC 0.736, 95% CI 0.673–0.817). At the optimal threshold of 9.9%, the positive predictive value (PPV) was 53.2% and the negative predictive value (NPV) was 84.4%. The optimal cutoff value determined by the Youden index was 9.9%, with sensitivity calculated as 65.4% and specificity as 78.0% (Figure 1). In subgroup analysis, the effect of CRL discordance was consistent in both MCDA twins (FGR rate 82.4% vs. 34.4%; OR 8.91, 95% CI 2.10–37.78, *p* = 0.001) and DCDA twins (OR 7.31, 95% CI 2.86–18.65, *p* < 0.001), but was higher in the monochorionic group. These findings are consistent with the decisive role of placental sharing dynamics in this population.

In the CRL discordance group, both the 1-min and 5-min Apgar scores were significantly lower. In Twin 1, the mean 1-min Apgar was 6.5 ± 1.9 versus 7.4 ± 1.5 (*p* = 0.003), and the 5-min Apgar was 8.1 ± 1.6 versus 8.6 ± 1.4 (*p* = 0.005). In Twin 2, the 1-min Apgar score was 6.6 ± 1.9 versus 7.3 ± 1.7 (*p* = 0.011), and the 5-min Apgar score was 8.1 ± 1.7 versus 8.5 ± 1.5 (*p* = 0.025). Regarding birth weight, Twin 2 had significantly lower mean birth weight in the CRL discordance ≥ 10% group compared with the CRL discordance < 10% group (1898 ± 583 g vs. 2119 ± 550 g, *p* = 0.023), whereas no significant difference was observed for Twin 1 (1912 ± 621 g vs. 2045 ± 548 g, *p* = 0.148). Cesarean delivery rate did not differ significantly between groups.

### 3.3. NT Discordance and Perinatal Outcomes

NT discordance ≥ 20% was associated with FGR (42.5% vs. 18.0%; univariate OR 3.36, 95% CI 1.72–6.57, *p* < 0.001; AUC 0.612; optimal cutoff value 21.4%, sensitivity 57.7%, specificity 70.8%). At the optimal threshold of 21.4%, the PPV was 42.5% and the NPV was 82.0%. In multivariate analysis, NT discordance ≥ 20% continued to be independently associated with the birth-weight-based FGR proxy (OR 3.74, 95% CI 1.91–8.39, *p* < 0.001); the addition of confounders did not significantly alter the effect size. NT discordance was not a significant predictor of preeclampsia in univariate (OR 1.23, *p* = 0.742) or multivariate analysis (*p* = 0.895). NT discordance was associated with a decrease in the 1-min Apgar score (6.7 ± 1.9 vs. 7.3 ± 1.7, *p* = 0.039) and birth weight (1.951 ± 614 g vs. 2.106 ± 532 g, *p* = 0.036) in Twin 2. Detailed findings are presented in Table 3.

### 3.4. Preeclampsia Predictors

In multivariate logistic regression, IVF conception was identified as the sole independent predictor of preeclampsia (OR 5.31, 95% CI 1.41–28.66, *p* = 0.016). Preeclampsia was observed in 13.6% (6/44) of IVF pregnancies, while the rate was 2.1% (3/140) in spontaneously conceived pregnancies (*p* = 0.004). CRL discordance (*p* = 0.119), NT discordance (*p* = 0.742), and chorionicity (*p* = 0.210) were not independently associated with preeclampsia. Multivariate regression results for FGR and preeclampsia are summarized in Table 4. Given the limited number of preeclampsia events, this model should be interpreted as exploratory.

## 4. Discussion

In this retrospective cohort study, first-trimester CRL discordance ≥ 10% was independently associated with the birth-weight-based FGR proxy in 184 consecutive twin pregnancies (adjusted OR 7.79; AUC 0.736). NT discordance ≥ 20% provided complementary but more modest predictive value (AUC 0.612). Neither CRL nor NT discordance was independently associated with preeclampsia; instead, IVF conception was the only independent predictor in the exploratory preeclampsia model (OR 5.31). Both discordance parameters were additionally associated with lower 1- and 5-min Apgar scores, extending their prognostic relevance beyond growth restriction.

The association between CRL discordance and FGR is biologically plausible. Early differences in placental perfusion arising during rapid trophoblast invasion (weeks 6–10) may be reflected in differential fetal growth trajectories at the 11–14-week scan. Litwinska and colleagues, in a large cohort of 6225 monochorionic twin pregnancies, showed that CRL discordance ≥ 10% predicted fetal loss and the need for laser surgery at 11–13 weeks [7]. A Danish cohort study of 762 MCDA pregnancies similarly identified CRL discordance ≥ 10% as the most important first-trimester predictor of severe birth weight discordance [12]. The present study extends these observations to a mixed-chorionicity cohort, with consistent effects observed in both DCDA (OR 7.31) and MCDA (OR 8.91) twins, albeit more pronounced in the monochorionic group [13,18,19].

When modelled as a continuous variable, each 1% increment in CRL discordance was independently associated with a 20% increase in the odds of FGR (OR = 1.20, 95% CI 1.13–1.32), consistent with a pattern of possible graded association rather than a mere threshold effect. NT discordance, by contrast, did not demonstrate a significant dose–response relationship (OR = 1.02, 95% CI 0.99–1.05). These findings suggest that early placental mass asymmetry, as captured by CRL, may more closely reflect early divergence in fetal growth pattern than nuchal fluid dynamics.

The optimal CRL discordance threshold identified in our cohort (9.9%) closely aligns with the widely accepted 10% cutoff [20], lending external validity to our ROC analysis, although prospective validation is still required before clinical implementation. This threshold is incorporated in current ISUOG twin pregnancy follow-up guidelines; in our cohort, pregnancies exceeding this threshold had a more than eightfold higher risk of FGR, suggesting that the 11–14-week scan may offer a potential opportunity for earlier risk stratification and individualized surveillance planning.

NT discordance ≥ 20% independently predicts FGR, but its discriminatory performance is more modest (AUC 0.612 vs. 0.736 for CRL). This finding is consistent with previous studies investigating the association of NT discordance, which has been suggested to reflect early cardiac function or placental perfusion differences between twins, with TTTS and adverse outcomes, which reported conflicting results [9,21]. Importantly, no evidence was found for an association between NT discordance and preeclampsia, despite suggestions from some small series [10]. This inconsistency is most likely due to our study having a sufficient sample size to adjust for stronger confounders such as IVF and chorionicity. By contrast, NT discordance appeared to provide weaker and less consistent prognostic information, suggesting that it may capture a related but less direct dimension of early placental or hemodynamic asymmetry. The absence of a significant continuous association further supports its role as a secondary rather than primary prognostic marker.

In contrast to its role in fetal growth prediction, neither CRL nor NT discordance showed independent predictive value for preeclampsia, which directs attention toward ART-related mechanisms. The independent association between IVF conception and preeclampsia (OR 5.31) is consistent with the growing literature linking ART with impaired trophoblast invasion and defective placentation [22]. Dai and colleagues’ large-scale study reported that IVF is an independent risk factor for preeclampsia in dichorionic twin pregnancies [23,24]; systematic reviews and meta-analyses have also confirmed approximately double the risk of preeclampsia associated with corpus luteum deficiency in frozen embryo transfer cycles. The development of preeclampsia in 13.6% of IVF-conceived twin pregnancies underscores the potential clinical significance of this finding. The shift in the direction of the IVF coefficient after multivariable adjustment for FGR most likely reflects confounding rather than a reproducible biological association. Given the limited number of preeclampsia events (*n* = 9), the multivariable preeclampsia model should be considered exploratory and interpreted with caution.

This study has several strengths. It was conducted at a single high-volume reference center using a cohort, standardized ultrasound protocols, and consistent outcome assessment, thereby minimizing measurement heterogeneity. All first-trimester ultrasound examinations were performed by certified operators using a single device (GE Voluson E8), thus reducing inter-operator measurement variability. Multivariate analysis controlled for the main confounders identified in the literature.

This study has several limitations that should be considered when interpreting the findings. First, the retrospective single-center design precludes standardization of clinical management decisions and may introduce residual confounding from variables not available for systematic extraction, including uterine artery Doppler pulsatility index, placental volume, and inter-observer variability in ultrasound measurements. Although all first-trimester examinations were performed by certified operators under a standardized protocol, the absence of prospective data collection limits causal inference. Second, the preeclampsia analysis is constrained by a low event count (*n* = 9), yielding an events-per-variable ratio below the conventional threshold of ten recommended for logistic regression. As a consequence, the odds ratio for IVF conception (OR 5.31, 95% CI 1.41–28.66), while statistically significant, carries wide confidence intervals and should be regarded as hypothesis-generating rather than confirmatory. Replication in larger, prospective cohorts with standardized preeclampsia ascertainment is required before these results can inform clinical screening algorithms. Third, FGR was operationally defined as birth weight below the 10th percentile using INTERGROWTH-21st newborn standards [16], a criterion that has been validated for use in resource-limited settings and enables reproducible retrospective extraction. However, contemporary consensus definitions of FGR additionally incorporate prenatal Doppler surveillance data to identify hemodynamically compromised fetuses prior to delivery [14]. Because umbilical and uterine artery Doppler indices were not available in a sufficiently complete and standardized form for retrospective analysis, our birth-weight-based definition may modestly underestimate true FGR prevalence and could introduce misclassification bias toward the null, potentially attenuating the observed effect sizes. Fourth, the study was conducted at a single tertiary-level obstetrics center in Ankara, Turkey, where the patient population is characterized by specific demographic and socioeconomic features. Accordingly, the external validity of the findings—particularly the optimal CRL discordance cutoff of 9.9%—should be evaluated prospectively in ethnically and institutionally diverse cohorts before implementation as a universal screening threshold. Fifth, to address the potential influence of chorionicity on the discordance–FGR association, interaction testing was performed. No significant interaction was observed between CRL discordance and chorionicity subgroup (likelihood ratio *p* = 0.564), supporting the consistency of the CRL–growth-restricted outcome association across both DCDA and MCDA pregnancies. For NT discordance, a nominally significant interaction term was identified (*p* = 0.020); however, this result should be interpreted with caution given the small number of NT-positive MCDA cases (*n* = 3) and model non-convergence, precluding reliable subgroup-specific estimates. Sixth, neonatal follow-up data beyond birth weight and Apgar scores were not systematically available for retrospective extraction, which limited the comprehensiveness of neonatal outcome assessment. Despite these limitations, the consistent biological gradient observed across both categorical and continuous models, the alignment of our cutoff with current ISUOG guidelines [20], and the mixed-chorionicity design collectively support the clinical relevance of these findings.

## 5. Conclusions

In twin pregnancies, first-trimester CRL discordance emerged as a stronger predictor of growth-restricted outcome than NT discordance. Continuous modelling further supported a dose–response relationship for CRL discordance (OR 1.20 per 1% increment). These findings suggest that intertwin CRL discordance may serve as an early marker of birth-weight-based adverse growth outcome; however, the retrospective design and moderate discriminatory performance indicate that prospective validation incorporating Doppler-based FGR criteria is required before clinical application. The preeclampsia findings, including the association with IVF conception, should be interpreted cautiously given the limited number of events.

## Figures and Tables

**Table 1:** Demographic and Clinical Characteristics of the Study Cohort (N = 184).

Variable	Total (N = 184)	CRL Discordance ≥ 10% (*n* = 62)	CRL Discordance < 10% (*n* = 122)
Age (years), mean ± SD	29.4 ± 6.2	29.1 ± 6.4	29.6 ± 6.1
BMI (kg/m^2^), mean ± SD	30.8 ± 5.4	31.3 ± 5.6	30.5 ± 5.2
Nulliparity, n (%)	104 (56.5)	35 (56.5)	69 (56.6)
IVF conception, n (%)	44 (23.9)	15 (24.2)	29 (23.8)
Chorionicity, n (%)			
Dichorionic–diamniotic	133 (72.3)	34 (54.8)	99 (81.1)
Monochorionic–diamniotic	49 (26.6)	17 (27.4)	32 (26.2)
Monochorionic–monoamniotic	2 (1.1)	0 (0.0)	2 (1.6)
Gestational age at scan (weeks)	12.3 ± 0.7	12.3 ± 0.8	12.2 ± 0.7
CRL discordance (%), mean ± SD	9.1 ± 7.8	18.7 ± 8.2	3.8 ± 2.6
NT discordance (%), mean ± SD	17.4 ± 14.1	22.9 ± 16.3	14.3 ± 12.0

BMI, body mass index; CRL, crown–rump length; IVF, in vitro fertilization; NT, nuchal translucency; SD, standard deviation.

**Table 2:** Perinatal Outcomes According to CRL Discordance Status.

Outcome	CRL Discordance < 10% (*n* = 122)	CRL Discordance ≥ 10% (*n* = 62)	Univariate OR (95% CI)/*p*
Pregnancy complications, n (%)			
FGR (≥1 twin)	19 (15.6)	33 (53.2)	6.57 (3.28–13.16)/<0.001
Preeclampsia	4 (3.3)	5 (8.1)	2.59 (0.67–10.04)/0.119
Gestational hypertension	10 (8.2)	7 (11.3)	1.42 (0.52–3.90)/0.490
TTTS	4 (3.3)	7 (11.3)	3.72 (1.07–12.92)/0.038
IUFD	0 (0.0)	1 (1.6)	—/0.338
Birth outcomes			
Gestational age at delivery (weeks), mean ± SD	34.2 ± 3.1	33.6 ± 3.4	—/0.212
Cesarean delivery, n (%)	98 (80.3)	54 (87.1)	1.65 (0.71–3.85)/0.247
Twin 1 results			
Birth weight (g), mean ± SD	2045 ± 548	1912 ± 621	—/0.148
Apgar score at 1 min, mean ± SD	7.4 ± 1.5	6.5 ± 1.9	—/0.003
Apgar score at 5 min, mean ± SD	8.6 ± 1.4	8.1 ± 1.6	—/0.005
Twin 2 results			
Birth weight (g), mean ± SD	2119 ± 550	1898 ± 583	—/0.023
Apgar score at 1 min, mean ± SD	7.3 ± 1.7	6.6 ± 1.9	—/0.011
Apgar score at 5 min, mean ± SD	8.5 ± 1.5	8.1 ± 1.7	—/0.025

CI, confidence interval; CRL, crown–rump length; IUFD, intrauterine fetal death; FGR, fetal growth restriction; OR, odds ratio; SD, standard deviation; TTTS, twin-to-twin transfusion syndrome.

**Table 3:** Perinatal Outcomes According to NT Discordance Status.

Outcome	NT Discordance < 20% (*n* = 111)	NT Discordance ≥ 20% (*n* = 73)	Univariate OR (95% CI)/*p*
Pregnancy complications, n (%)			
FGR (≥1 twin)	21 (18.0)	31 (42.5)	3.36 (1.72–6.57)/<0.001
Preeclampsia	5 (4.5)	4 (5.5)	1.23 (0.32–4.70)/0.742
Gestational hypertension	9 (8.1)	8 (11.0)	1.40 (0.52–3.77)/0.507
TTTS	6 (5.4)	5 (6.8)	1.29 (0.38–4.39)/0.686
IUFD	1 (0.9)	1 (1.4)	1.54 (0.09–25.14)/1.000
Birth outcomes			
Gestational age at delivery (weeks), mean ± SD	34.3 ± 3.0	33.7 ± 3.5	—/0.256
Cesarean delivery, n (%)	90 (81.1)	62 (84.9)	1.32 (0.59–2.94)/0.495
Twin 1 results			
Birth weight (g), mean ± SD	2036 ± 561	1956 ± 609	—/0.382
Apgar score at 1 min, mean ± SD	7.2 ± 1.6	6.9 ± 1.9	—/0.285
Apgar score at 5 min, mean ± SD	8.5 ± 1.4	8.3 ± 1.6	—/0.412
Twin 2 results			
Birth weight (g), mean ± SD	2106 ± 532	1951 ± 614	—/0.036
Apgar score at 1 min, mean ± SD	7.3 ± 1.7	6.7 ± 1.9	—/0.039
Apgar score at 5 min, mean ± SD	8.5 ± 1.5	8.2 ± 1.7	—/0.187

CI, confidence interval; IUFD, intrauterine fetal death; FGR, fetal growth restriction; NT, nuchal translucency; OR, odds ratio; SD, standard deviation; TTTS, twin-to-twin transfusion syndrome.

**Table 4:** Multivariate Logistic Regression Analysis for FGR and Preeclampsia.

Variable	Univariate OR	95% CI	Adjusted OR	95% CI/*p*
Result: FGR (≥1 twin)—Model 1: CRL discordance				
CRL discordance ≥ 10%	6.57	3.28–13.16	7.79	3.95–20.12/<0.001
CRL discordance (per 1%)	—	—	1.20	1.13–1.32/<0.001
Monochorionicity	3.98	2.01–7.88	4.38	1.88–10.22/<0.001
BMI (per kg/m^2^)	1.04	0.98–1.10	1.03	0.97–1.09/0.312
IVF conception	0.48	0.21–1.13	1.22	0.56–2.67/0.124
Result: FGR (≥1 twin)—Model 2: NT discordance				
NT discordance ≥ 20%	3.36	1.72–6.57	3.74	1.91–8.39/<0.001
NT discordance (per 1%)	—	—	1.02	0.99–1.05/0.248
Monochorionicity	3.98	2.01–7.88	3.91	1.73–8.84/0.001
BMI (per kg/m^2^)	1.04	0.98–1.10	1.03	0.97–1.09/0.348
IVF conception	0.48	0.21–1.13	1.19	0.55–2.60/0.124
Result: Preeclampsia—Model 3: All candidate variables				
IVF conception	4.28	1.07–17.13	5.31	1.41–28.66/0.016
CRL discordance ≥ 10%	2.59	0.67–10.04	1.98	0.44–8.93/0.374
NT discordance ≥ 20%	1.23	0.32–4.70	1.09	0.27–4.44/0.895
Monochorionicity	1.44	0.37–5.54	1.38	0.32–5.92/0.667
BMI (per kg/m^2^)	1.08	0.98–1.19	1.06	0.95–1.18/0.308

Note: Due to the multicollinearity between CRL and NT discordance, separate models (Model 1 and 2) were created. Model 3 also includes NT discordance, which previous literature has suggested may be associated with preeclampsia. BMI, body mass index; CRL, crown–rump length; CI, confidence interval; FGR, fetal growth restriction; IVF, in vitro fertilization; NT, nuchal translucency; OR, odds ratio. Due to multicollinearity between CRL and NT discordance, separate multivariable models were constructed for these markers. The reversal in the direction of the IVF coefficient after adjustment likely reflects confounding by chorionicity and maternal characteristics rather than a stable independent association with fetal growth restriction. The preeclampsia model should be interpreted as exploratory because of the limited number of events (*n* = 9).

## Data Availability

The datasets used and analyzed during this study are available from the corresponding author upon reasonable request, subject to applicable privacy and ethical restrictions.

## Supplementary Materials

The following supporting information can be downloaded at: https://www.mdpi.com/article/10.3390/jcm15083129/s1, Table S1: STROBE Checklist—Cohort Study.

## Abbreviations

The following abbreviations are used in this manuscript: AUC, area under the curve; BMI, body mass index; CI, confidence interval; CRL, crown–rump length; DCDA, dichorionic–diamniotic; FGR, fetal growth restriction; IVF, in vitro fertilization; IUFD, intrauterine fetal death; MCDA, monochorionic–diamniotic; MCMA, monochorionic–monoamniotic; NT, nuchal translucency; OR, odds ratio; ROC, receiver operating characteristic; SD, standard deviation; TTTS, twin-to-twin transfusion syndrome.
