# Supplementary material for: Comparative Predictive Value of First-Trimester Crown–Rump Length and Nuchal Translucency Discordance for Fetal Growth Restriction in Twin Pregnancies: A Retrospective Cohort Study
Source: J Clin Med. 2026 Apr 20;15(8):3129. doi: 10.3390/jcm15083129 (PMC13116854; doi:10.3390/jcm15083129)
Supplement: Supplementary file 1 [file jcm-15-03129-s001.zip › jcm-4220119-supplementary.pdf]

STROBE Checklist — Cohort Study

Manuscript: Comparative Predictive Value of First-Trimester CRL and NT Discordance for FGR in Twin Pregnancies

Journal: Journal of Clinical Medicine (MDPI) | Corresponding Author: Cansın Eroğlu

N/A = not applicable to this study design.

Item	Recommendation	Location in Manuscript
1a	Indicate the study's design with a commonly used term in the title or the abstract	Title, Abstract
1b	Provide in the abstract an informative and balanced summary of what was done and what was found	Abstract
2	Explain the scientific background and rationale for the investigation being reported	Introduction
3	State specific objectives, including any prespecified hypotheses	Introduction
4	Present key elements of study design early in the paper	2.1
5	Describe the setting, locations, and relevant dates, including periods of recruitment, exposure, follow-up, and data collection	2.1
6	Give the eligibility criteria, and the sources and methods of selection of participants. Describe methods of follow-up	2.1
7	Clearly define all outcomes, exposures, predictors, potential confounders, and effect modifiers. Give diagnostic criteria, if applicable	2.2, 2.3
8	For each variable of interest, give sources of data and details of methods of assessment (measurement)	2.2
9	Describe any efforts to address potential sources of bias	2.1, Discussion
10	Explain how the study size was arrived at	2.1
11	Explain how quantitative variables were handled in the analyses	2.4
12a	Describe all statistical methods, including those used to control for confounding	2.4
12b	Describe any methods used to examine subgroups and interactions	2.4, 3.2
12c	Explain how missing data were addressed	2.1
12d	Explain how loss to follow-up was addressed	N/A
12e	Describe any sensitivity analyses	N/A
13a	Report numbers of individuals at each stage of study	3.1
13b	Give reasons for non-participation at each stage	3.1
13c	Consider use of a flow diagram	3.1 (narrative)
14a	Give characteristics of study participants and information on exposures and potential confounders	Table 1
14b	Indicate number of participants with missing data for each variable of interest	N/A
14c	Summarise follow-up time	N/A
15	Report numbers of outcome events or summary measures over time	3.2, 3.3, 3.4
16a	Give unadjusted and confounder-adjusted estimates and their precision (95% CI)	Tables 2–4

Item	Recommendation	Location in Manuscript
16b	Report category boundaries when continuous variables were categorized	2.3, 2.4
16c	If relevant, consider translating estimates of relative risk into absolute risk	N/A
17	Report other analyses done — subgroups, interactions, sensitivity analyses	3.2
18	Summarise key results with reference to study objectives	Discussion
19	Discuss limitations of the study, taking into account sources of potential bias or imprecision	Limitations
20	Give a cautious overall interpretation of results	Discussion
21	Discuss the generalisability (external validity) of the study results	Limitations
22	Give the source of funding and the role of the funders	Funding
